# Dihydroartemisinin Promotes N1 Polarization of Tumor-Associated Neutrophils and Enhances Their Anti-Tumor Activity via Hub Gene Modulation

**DOI:** 10.3390/ph19010088

**Published:** 2026-01-01

**Authors:** Wenjia Guo, Yu’e Liu, Wencong Ma, Jinghan Wang, Bingdi Chen, Lieying Fan

**Affiliations:** 1 Department of Laboratory Medicine, Shanghai East Hospital, School of Medicine, Tongji University, Shanghai 200092, China; 2 Department of Hematology/Oncology, Boston Children’s Hospital, Harvard Medical School, Boston, MA 02115, USA; 3 Institute of Hepatobiliary and Pancreatic Surgery, Department of Hepatobiliary and Pancreatic Surgery, Shanghai East Hospital, School of Medicine, Tongji University, Shanghai 200092, China; wencongsmmu2007@163.com (W.M.);; 4 The Institute for Biomedical Engineering and Nano Science, School of Medicine, Tongji University, Shanghai 200092, China

**Keywords:** dihydroartemisinin, tumor-associated neutrophils, molecular docking, polarization

## Abstract

**Background**: Tumor-associated neutrophils (TANs) exhibit remarkable functional plasticity within tumor microenvironment (TME), with N1-like subtypes promoting anti-tumor immunity and N2-like subtypes facilitating tumor progression. Despite their critical role in cancer immunology, strategies to selectively modulate TAN polarization remain limited. **Methods**: We integrated transcriptomic analyses of TAN subtypes to identify potential hub molecules. Molecular docking and experimental assays were used to evaluate DHA’s effect on neutrophil-like cell polarization. **Results**: Hub genes (*TNF*, *IL1B*, *PTGS2*, *BCL2A1*, *MSR1*, *ACOD1*, *CXCL16*, *CLEC10A*, and *SOCS3*) were identified, with TNF serving as a potential core regulator. Molecular docking indicated that DHA forms stable interactions hub proteins. Experimentally, DHA treatment of neutrophil-like dNB4 cells promoted N1 polarization, evidenced by upregulation of *TNF, IL1B, PTGS2, BCL2A1, MSR1, ACOD1, CXCL16*, and N1 markers *PD-L1* and *NOX2*, and downregulation of N2 marker *CEACAM8* and hub genes *CLEC10A* and *SOCS3*. Functional assays demonstrated that DHA-treated cells exhibited increased secretion of TNF, IL1β, ROS, and PD-L1, accompanied by enhanced cytotoxic activity against hepatocellular carcinoma cells in a co-culture system. **Conclusions**: These findings reveal the molecular mechanisms underlying TAN polarization, and establish DHA as a potent immunomodulatory agent capable of reshaping TANs toward an anti-tumor phenotype.

## 1. Introduction

The concept of the tumor microenvironment (TME) stems from Paget’s “seed and soil” hypothesis, first proposed in 1889 [1], referring to a specialized local milieu composed of diverse cellular components, extracellular matrix, and bioactive molecules. This conceptual framework shifts the focus from intrinsic tumorigenic mechanisms to the dynamic interplay between cancer cells and their surrounding stromal ecosystem. The TME is a specialized local milieu composed of diverse cellular components, extracellular matrix, and bioactive molecules, shifting the focus from intrinsic tumorigenic mechanisms to the dynamic interplay between cancer cells and their surrounding stromal ecosystem. Accumulating evidence suggests that the TME resembles a state of chronic, non-resolving inflammation, with inflammatory responses playing a pivotal role in tumor progression [2,3,4]. Within this context, tumor-associated neutrophils (TANs)—the most abundant innate immune cell population in the TME—exhibit functional plasticity that contrasts sharply with the protective, pro-inflammatory roles of neutrophils in acute infection or tissue injury. Rather than exerting anti-tumor activity, certain TAN subtypes acquire pro-tumorigenic properties, contributing to immune evasion, angiogenesis, and metastasis [5,6,7,8]. Building on these observations, Fridlender et al. proposed a functional dichotomy of TANs into N1 and N2 phenotypes based on their distinct immunomodulatory profiles [9]. The N1 phenotype is characterized by the secretion of pro-inflammatory mediators such as IL-12, CCL3, CXCL9, CXCL10, and TNF-α, which promote the recruitment and activation of cytotoxic CD8^+^ T cells, thereby enhancing anti-tumor immunity [5,6,10]. In contrast, the N2 phenotype is marked by high expression of CXCR2 and the production of IL-8, CCL2, CCL5, neutrophil elastase, cathepsin G, arginase, matrix metalloproteinases, and pro-angiogenic factors. These factors collectively facilitate tumor angiogenesis, invasion, and metastasis [5,9,11,12], while simultaneously suppressing T cell proliferation and cytokine production, promoting regulatory T cell expansion, and reinforcing immune tolerance toward tumor cells. The polarization of TANs is dynamically regulated by key soluble factors within the TME, including TGF-β, interferon-γ (IFN-γ), granulocyte colony-stimulating factor (G-CSF), and hypoxic conditions [9,10,13,14,15,16]. Specifically, signaling via TGF-βor G-CSF drives TAN polarization toward the N2 phenotype, whereas inhibition of the TGF-β pathway or type I interferon signaling promotes a shift toward the N1 phenotype [5,9,17,18,19].

TANs represent a promising therapeutic target in cancer immunotherapy. Current strategies primarily focus on modulating TANs’ function, such as inhibiting cytokines involved in their recruitment and polarization, or selectively disrupting the pro-tumorigenic activities of N2. Notably, N2 subtype can be reprogrammed to adopt an anti-tumor N1 phenotype in response to IFN-γ and TNF-α [20]. It is important to emphasize that neutrophils are a critical component of the innate immune system in host defense against infections; therefore, excessive depletion or suppression may disrupt immune homeostasis. Consequently, therapeutic approaches should aim to reshape the functional profile of TANs rather than eliminate them entirely [21,22].

In this context, traditional Chinese medicine has emerged as a promising source of immunomodulatory agents. For instance, Chen et al. demonstrated that the extract of Yi Qi Chu Formula suppresses lung tumor growth in C57 mice, accompanied by increased infiltration of CD4^+^ and CD8^+^ T cells, enhanced dendritic cell maturation, and a favorable shift in the M1/M2 ratio of tumor-associated macrophages [23]. Likewise, dihydroartemisinin (DHA) has been shown to increase intra-tumoral CD8^+^ T cell populations in hepatocellular carcinoma models and expand functional PD-1^−^ CD4^+^ T cells in the spleen [24]. Collectively, these findings suggest that herbal compounds may offer a potent strategy to address this therapeutic challenge.

In addition to its effects on malignant diseases, DHA has garnered significant attention for its immunomodulatory properties in recent years. Wang et al. observed in a murine colon cancer model that this semi-synthetic derivative promotes the proliferation of Tregs and facilitates the differentiation of Th1 and Th17 cells [25]. They therefore hypothesized that DHA may attenuate intestinal inflammation and inhibit the progression of colon cancer by restoring the balance between Th17 and Treg cells, given that Th17 cells secrete pro-inflammatory cytokines such as IL-17 and IL-22, whereas Treg cells maintain immune tolerance through the secretion of immunosuppressive cytokines including IL-10 and TGF-β [26]. Furthermore, Yu et al. confirmed that DHA increases the infiltration of CD8^+^ T cells in the TME and enhances anti-tumor immunity in melanoma-bearing mice [27]. Additionally, Zhao et al. demonstrated that the regulatory effect of DHA on the number and function of Tregs is mediated via the TGF-βR/Smad signaling pathway and does not lead to systemic immunosuppression in an experimental autoimmune encephalomyelitis mouse model [28]. According to Christoph et al., DHA potentiates reactive oxygen species (ROS) production in neutrophils [29]. Based on these findings, we propose the hypothesis that DHA modulates TANs phenotypic polarization—potentially toward an N1-like anti-tumor subtype—and thereby enhances their cytotoxic capacity against cancer cells.

## 2. Results

### 2.1. Transcriptomic Profiling and Functional Enrichment of N1 and N2 TANs

**Figure 1:**
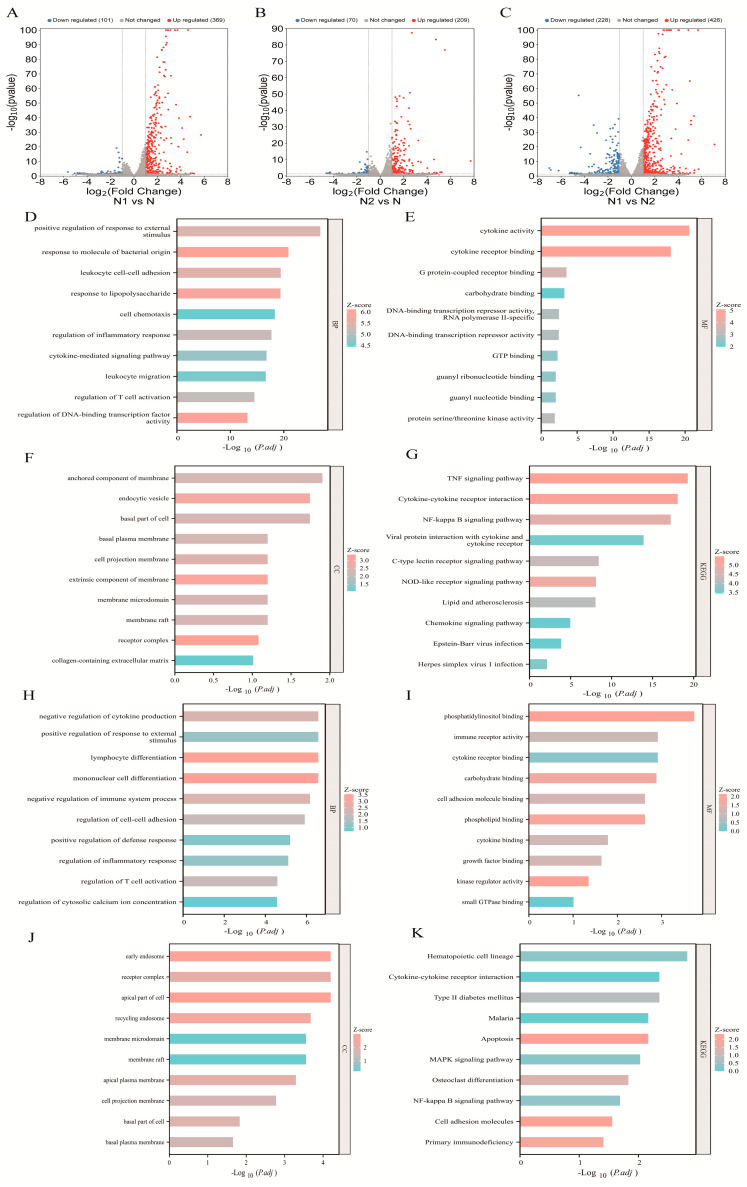
Bioinformatic analysis of E-MTAB-1050. (**A**–**C**) Volcano plots of DEGs between the N1 vs. N, N2 vs. N and N1 vs. N2 subtypes. (**D**–**G**) GO biological processes, molecular functions, cellular components, and KEGG pathway analysis of DEGs between the N1 and N subtypes; (**H**–**K**) GO biological processes, molecular functions, cellular components, and KEGG pathway analysis of DEGs between the N2 and N subtypes.

To investigate the transcriptomic differences among TAN subtypes, we analyzed dataset E-MTAB-1050. N1 cells were treated with 100 ng/mL LPS for 2 h, N2 cells with 20 ng/mL IL-4 for 2 h, and untreated neutrophils (N) served as controls. Differential expression analysis revealed 369 upregulated and 101 downregulated genes in N1 compared with N, while N2 exhibited 209 upregulated and 70 downregulated genes relative to N. Direct comparison between N1 and N2 identified 426 upregulated and 228 downregulated genes (Figure 1A–C).

Gene ontology (GO) analysis was performed to explore the functional roles of DEGs in TAN subtypes. In N1 cells, DEGs were significantly enriched in biological processes related to responses to exogenous stimuli, pathogen-associated molecular patterns, and T cell activation, indicating successful polarization toward the N1 phenotype. Molecular function analysis further revealed upregulation of genes involved in adhesion, migration, chemotaxis, and cytokine production, with enrichment in infection- and inflammation-related pathways, including TNF, NF-κB, and NOD-like receptor signaling (Figure 1D–G).

In contrast, DEGs in N2 cells were predominantly associated with metabolic processes, such as calcium signaling, phospholipid, and phosphatidylinositol metabolism (Figure 1H–J). Subcellular localization analysis showed that most DEGs were localized to the basal membrane, receptor complexes, and cellular protrusions (Figure 1F, K). Collectively, these results demonstrate distinct transcriptional programs and functional divergence between N1 and N2 TAN subtypes.

### 2.2. Network-Based Identification of Hub Genes in TAN Subtypes

**Figure 2:**
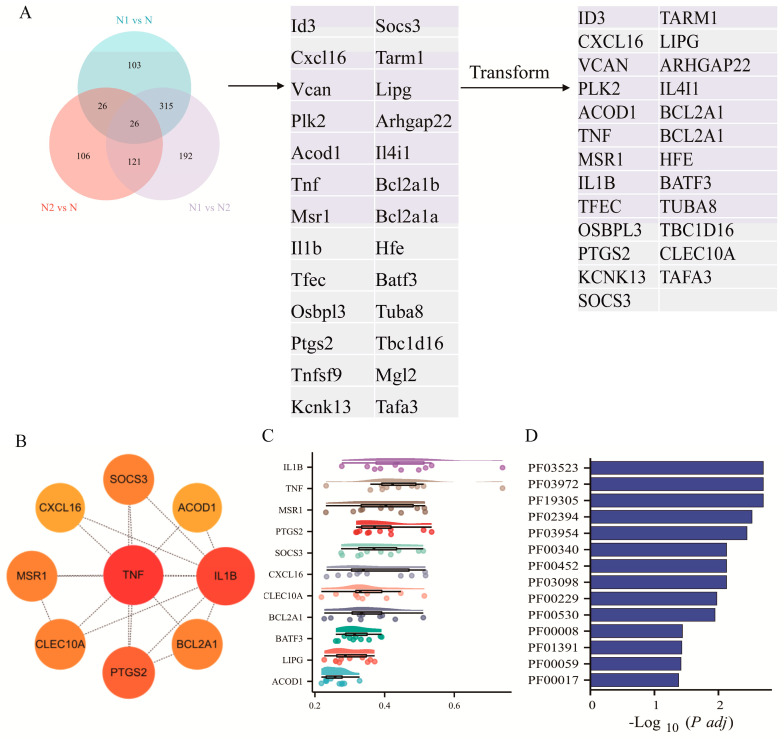
Enrichment analysis of hub genes of TANs. (**A**) Venn diagram showing DEGs across TAN subgroups and human orthologous genes. (**B**) Hub genes identified from the integrated DEG network. (**C**) Friends enrichment analysis of hub genes. (**D**) Domain enrichment analysis of hub genes.

To identify key regulators underlying TAN subtype polarization, we first compared DEGs across N1 vs. N, N2 vs. N, and N1 vs. N2. A Venn diagram revealed 26 overlapping genes, of which 25 human orthologs were retained for further analysis (Figure 2A). Using the MCC algorithm, nine genes—*TNF, IL1B, PTGS2, BCL2A1, MSR1, ACOD1, CXCL16, CLEC10A*, and *SOCS3*—were identified as top hub genes. Among these, *TNF, IL1B, PTGS2, BCL2A1, MSR1, ACOD1*, and *CXCL16* exhibited higher expression in N1, whereas *CLEC10A* and *SOCS3* were predominantly upregulated in N2 (Figure 2B).

Network connectivity analysis (Friends analysis) indicated that *IL1B* and *TNF* function as central nodes, displaying the highest average connectivity scores of 0.474 and 0.462, respectively, suggesting their potential role as master regulators of TAN polarization. Furthermore, Pfam domain enrichment analysis revealed seven significantly enriched domains—PF03523, PF03972, PF19305, PF02394, PF00452, PF03098, and PF00229 (*p* < 0.05)—among the nine hub genes, indicating the presence of conserved structural motifs likely contributing to their biological functions (Figure 2C, D).

### 2.3. Molecular Docking Analysis of Hub Genes with DHA

**Figure 3:**
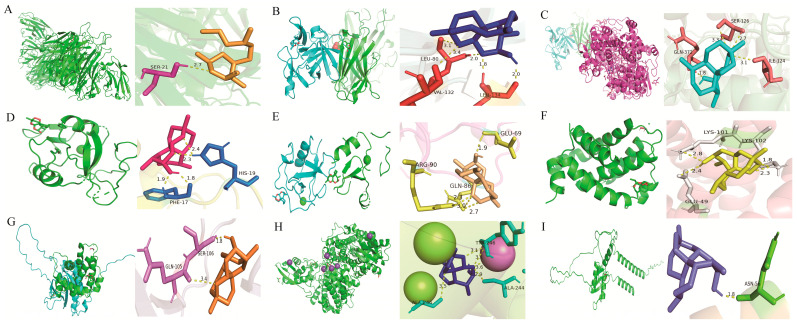
Molecular docking conformations of DHA with hub genes. (**A**–**I**) Predicted binding modes between DHA and the protein products of TNF, IL1B, PTGS2, CLEC10A, MSR1, BCL2A1, SOCS3, ACOD1 and CXCL16, respectively.

To explore the potential molecular interactions between DHA and the identified hub genes, in silico molecular docking analyses were performed. Each hub gene-encoded protein structure was modeled and docked with DHA to evaluate binding affinities, predict interaction conformations, and identify putative binding sites. The docking results demonstrated that DHA formed stable interactions with hub proteins, suggesting potential direct modulatory effects on their biological functions. Notably, CXCL16 exhibited a relatively weaker binding affinity with a calculated binding energy of −4.96 kcal/mol (Table 1, Figure 3).

### 2.4. MD Analysis of TNF with DHA

**Figure 4:**
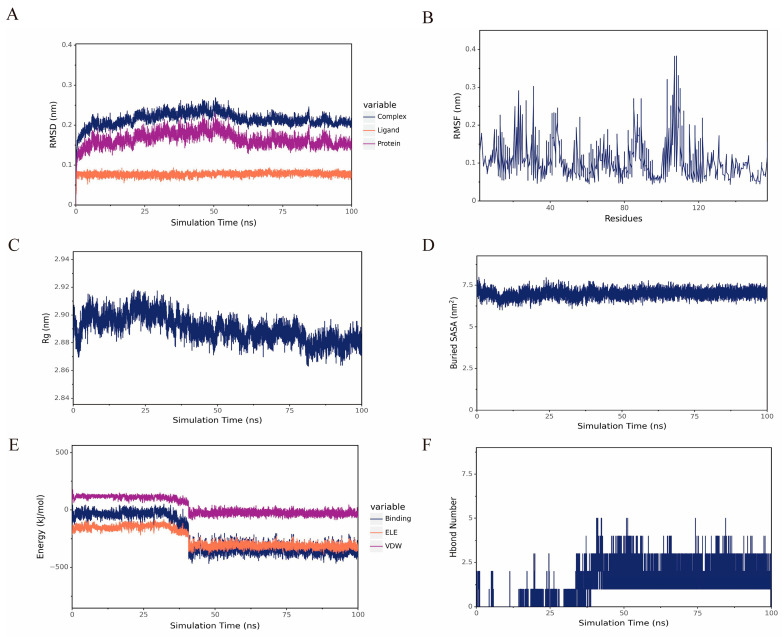
(**A**) RMSD of DHA and TNF. (**B**) RMSF of the protein in the complex. (**C**,**D**) Rg and Buried SASA between DHA and TNF of the complex. (**E**) Van der Waals and electrostatic contributions to the binding energy between small molecules and proteins. (**F**) Number of hydrogen bonds.

The hub gene TNF was selected for further molecular dynamics simulation analysis. RMSD of the complex stabilized, indicating maintenance of structural integrity and stable ligand–protein binding throughout this phase. The radius of gyration (Rg) of the complex remains low and stable, indicating maintenance of a compact conformation and overall structural stability, supported by Buried SASA. According to the RMSF analysis, residues 100–110 may represent a potential specific site of action for DHA (Figure 4).

### 2.5. Dihydroartemisinin Polarized Neutrophil-like Cells Towards N1 Subtype

**Figure 5:**
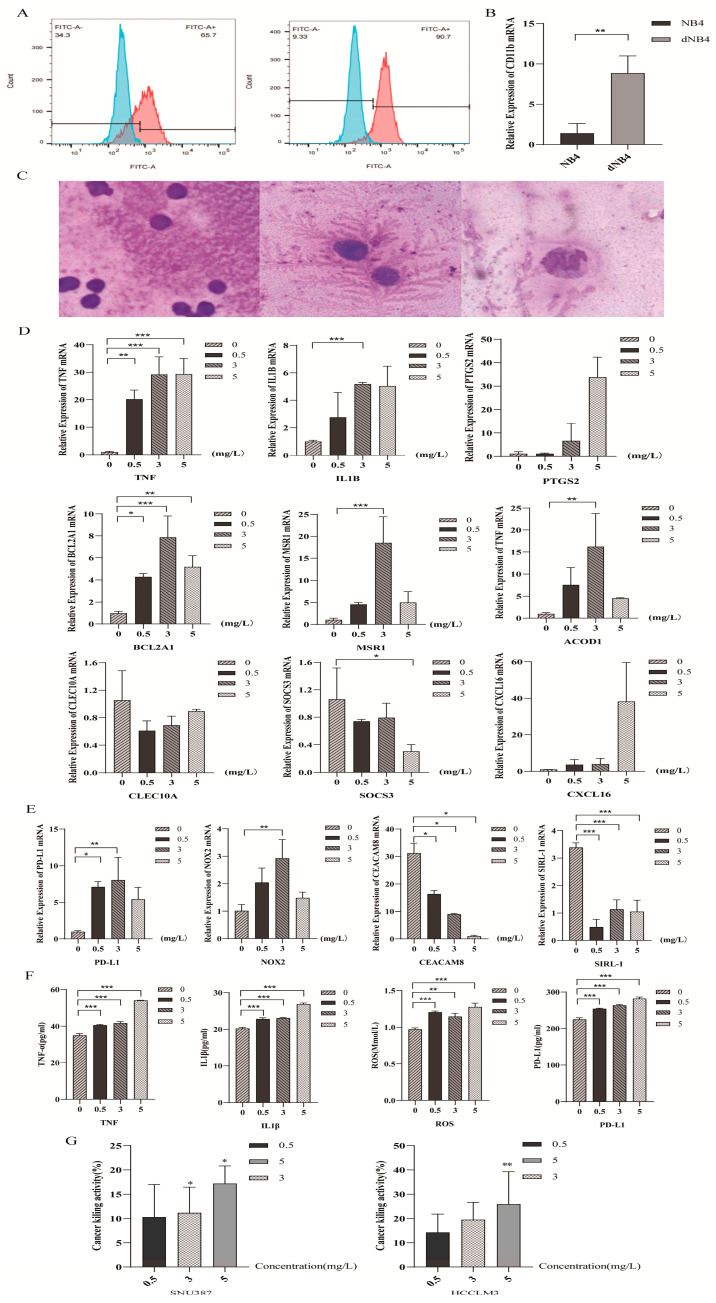
(**A**) Flow cytometric analysis of CD11b expression following ATRA-induced differentiation. (**B**) qRT-PCR indicating *CD11b* mRNA levels after ATRA induction. (**C**) Wright’s staining of morphological changes in dNB4 cells (scale bar = 50 µm, 1000×). (**D**) qRT-PCR of hub genes including *TNF, IL1B, PTGS2, BCL2A1, MSR1, ACOD1, CXCL16, CLEC10A* and *SOCS3* in DHA-treated dNB4 cells. (**E**) qRT-PCR of markers of N1 as *PD-L1, NOX2, CEACAM8,* and *SIRL-1* in DHA-treated dNB4 cells. (**F**) ELISA of TNF, IL-1β, ROS, and PD-L1 in DHA-treated dNB4 cell supernatants. (**G**) CKA evaluation of the tumor-killing capacity of DHA-treated dNB4 cells in a direct co-culture system. *p < 0.05, **p < 0.01, ***p < 0.001.

To investigate the effect of DHA on neutrophil polarization, NB4 cells were first differentiated into neutrophil-like cells using ATRA. Successful differentiation was confirmed by flow cytometry, with approximately 90% of cells expressing the neutrophil marker CD11b (*p* < 0.05). Morphological assessment using Wright’s staining revealed characteristic changes, including enlarged cell size, purplish-red cytoplasm, and lobulated, rod-shaped nuclei (Figure 5A–C).

dNB4 cells were subsequently treated with DHA at concentrations of 0.5, 3, and 5 mg/L for 4 h. qRT-PCR analysis demonstrated a concentration-dependent upregulation of N1-associated hub genes, including *TNF, IL1B, PTGS2, BCL2A1, MSR1, ACOD1, and CXCL16,* whereas N2-associated genes *CLEC10A* and *SOCS3* were downregulated (*p* < 0.05) (Figure 5D). Consistently, expression of N1 phenotypic markers *PD-L1* and *NOX2* was significantly increased, while the N2 marker CEACAM8 was suppressed (*p* < 0.05) (Figure 5E). ELISA measurements further confirmed elevated secretion of TNF, IL-1β, ROS, and PD-L1 in a dose-dependent manner (*p* < 0.05) (Figure 5F).

Functional validation using a co-culture killing assay (CKA) revealed that DHA-pretreated dNB4 cells exhibited enhanced cytotoxicity against liver cancer cells at a 1:3 effector-to-target ratio over 24 h. Notably, the anti-tumor effect increased with DHA concentration, supporting a dose-dependent enhancement of cytotoxic function (*p* < 0.05) (Figure 5G).

Collectively, these results demonstrate that DHA owns the potential of promoting the polarization of neutrophil-like cells toward an N1-like, anti-tumor phenotype, upregulating pro-inflammatory and cytotoxic genes, enhancing functional markers of N1 TANs, and increasing tumoricidal activity, highlighting its potential as an immunomodulatory agent in cancer therapy.

## 3. Discussion

In this study, we integrated transcriptomic analysis with functional validation to elucidate the molecular determinants of TAN polarization and assess the immunomodulatory effects of DHA. Analysis of the E-MTAB-1050 dataset identified distinct transcriptomic landscapes of N1 and N2 TANs, with N1 enriched for genes involved in host defense, cytokine secretion, and immune activation, whereas N2 exhibited signatures associated with immunosuppression and TME remodeling. Among the identified hub genes, *TNF* emerged as a central regulator, potentially orchestrating the phenotypic transition between TAN subtypes. Domain enrichment analysis further suggested that conserved functional motifs, including peroxidase-like and scavenger receptor domains, may underlie the responsiveness of TANs to pharmacological modulation.

This study identifies the hub genes of TANs through transcriptomic dataset analysis and experimentally validates that DHA promotes the polarization of dNB4 cells toward the N1 phenotype, providing novel insights into the molecular mechanisms underlying TAN polarization and the immunomodulatory effects of DHA.

Dataset E-MTAB-1050 utilized neutrophils isolated from the bone marrow of C57BL/6J mice, which have not encountered stimuli and therefore an ideal design for exploring neutrophil polarization. GO and KEGG analysis revealed that N1 exhibits enhanced capabilities in host defense, cell adhesion, cytokine secretion, and immune activation; upregulated genes are closely associated with inflammatory responses, among which *IL23A* showed the highest variation (7-fold increase). In contrast, functional profile of N2 suggests an immunosuppressive role, indicating their potential involvement in TME remodeling and modulation. Notably, DEGs from both subtypes were significantly enriched in cytokine–receptor interaction pathways, underscoring the critical role of soluble mediators within the TME as key extrinsic regulators of TANs’ polarization.

Given that multiple genes are involved in this process [30,31], suggesting coordinated protein activity, we focused on identifying the most biologically central and functionally interconnected hub genes. To achieve this, we integrated DEGs, PPIs, and the MCC algorithm—approaches well-suited for uncovering key regulatory nodes with high connectivity and functional significance. Nine key candidates: *TNF, IL1B, PTGS2, BCL2A1, MSR1, ACOD1, CXCL16, CLEC10A,* and *SOCS3* were yielded. Among these, *TNF* emerged as a pivotal gene, with Friends analysis revealing it to have the highest average similarity score across the network, suggesting that *TNF* may serve as a potential master regulator in TAN subtype transition. Furthermore, domain enrichment analysis via the Pfam database revealed that key hub genes are enriched in domains including macrophage scavenger receptor (PF03523), interleukin-1 precursor (PF02394), interleukin-1/18 (PF00340), tumor necrosis factor (PF00229) family, and apoptosis regulatory protein (PF00452). More importantly, PF03098 (animal heme peroxidase) suggested a potential role of the peroxidase-like structure in mediating DHA’s effects on neutrophils. These specific structural domains may serve as promising targets for developing TAN-directed therapeutic agents. Collectively, these findings indicate that TANs’ plasticity is dynamically regulated externally, with *TNF* acting as the core governor potentially, providing a novel and comprehensive perspective towards biomarkers for TAN subtypes.

Furthermore, molecular docking results between hub genes and DHA demonstrated that stable complexes were formed with all hub genes except *CXCL16*. Since dNB4-derived neutrophil model is widely adopted in neutrophil research due to its cost-effectiveness and experimental reproducibility, it was chosen to offer a controlled and consistent platform for recapitulating neutrophil responses under defined experimental conditions [32]. Based on literature indicating that the plasma concentration range of artemisinin-based drugs falls within 0.1–3 mg/L [29], dNB4 cells were treated with DHA at concentrations of 0.5, 3, and 5 mg/L for 4 h to simulate therapeutic exposure. qRT-PCR analysis revealed that the expression of *TNF, IL1B, PTGS2, BCL2A1, MSR1, ACOD1,* and *CXCL16* was upregulated in a concentration-dependent manner, whereas *CLEC10A* and *SOCS3* were downregulated, consistent with transcriptomic data. Notably, the expression levels of *BCL2A1, ACOD1,* and *MSR1* decreased at the 5 mg/L dose, which may be attributed to oxidative stress-induced apoptosis under high-dose DHA exposure. Similarly, DHA significantly increased the expression of N1-associated markers *PD-L1* and *NOX2*, while suppressing the expression of the N2 marker *CEACAM8* (*CD66b*). *NOX2* —key enzyme for ROS generation—indicated its involvement in regulating neutrophil adhesion, differentiation, and apoptosis. *CEACAM8* is widely recognized as a reliable in situ marker for N2, and multiple studies have demonstrated that elevated infiltration of CD66b^+^ TANs correlates with poor prognosis in various cancers [33]. For instance, Youjing Sheng et al. analyzed 302 breast cancer specimens by immunohistochemistry and reported that CD66b^+^ TAN infiltration was significantly correlated with shorter progression-free survival, supporting its utility as a predictive biomarker for metastasis and clinical outcome [31]. Similarly, Yuta Yamada et al. demonstrated that higher densities of CD66b+ TANs were positively associated with larger tumor size, lymph node and distant metastasis, S-phase fraction, and venous invasion, and further established CD66b+ TANs density as an independent prognostic factor for overall survival [34]. Next, ELISA further confirmed that DHA enhanced the secretion of TNF, IL-1β, ROS, and PD-L1 from dNB4 cells in a dose-dependent manner, supporting the transcriptional findings. Cytotoxicity was evaluated using the CKA, a refined in vitro method involving direct co-culture of leukocytes with adherent tumor cells for 12–24 h, followed by quantification of residual tumor cells to assess cytotoxic effects. An effector-to-target ratio of approximately 3:1 was determined to be optimal [35]. The CCK-8 assay demonstrated that DHA significantly enhanced the tumor-killing activity of dNB4 cells in a concentration-dependent trend. Collectively, these findings strongly support the role of DHA in promoting N1 polarization and enhancing their anti-tumor functionality.

Previous studies have demonstrated that artemisinin-based drugs enhance ROS release from neutrophils, alleviating their chemotaxis suppression as well as immune function in sepsis [29,36]. Within the context of tumor immunity, these compounds predominantly enhance immune responses. Youn Kyung Houh et al. demonstrated that artemisinin pretreatment enhances cytotoxic and degranulation activities of NK-92MI cells in a dose-dependent manner [37]. Ran Yu et al. reported that DHA treatment downregulates IL-10 and IL-6, upregulates IFN-γ, promotes splenic CD8^+^ T cell infiltration, and suppresses the activity of CD4^+^CD25^+^Foxp3^+^ regulatory T cells and IL-10^+^CD4^+^CD25^+^ T cells, thereby augmenting T cell-mediated antitumor immunity [27]. Xiao Chen et al. showed that artemisinin upregulates miR-142 expression and downregulates SIRT1, driving the differentiation of CD4^+^ T cells toward the Th1 phenotype and increasing apoptosis in ovarian cancer cells [38]. Taken together, DHA holds significant promise for modulating immune responses, offering broad therapeutic potential in disorders.

Despite these advances, several limitations warrant consideration. First, granulocyte heterogeneity and susceptibility to environmental influences suggest that validation using primary neutrophils is needed. Primary neutrophils, particularly TANs, represent a more physiologically relevant model for studying. Second, in vivo studies are required to fully assess the immunomodulatory effects of DHA within the complex tumor microenvironment. Finally, complementary techniques such as surface plasmon resonance, or cellular thermal shift assays could further clarify the direct interactions between DHA and hub gene products. For MD analysis, extended simulation duration as well as refined simulation parameters are required to enhance accuracy and sampling convergence. Moreover, the functional role of the potential core module gene *TNF* and its underlying mechanisms require further validation through in vivo studies, and the use of bioactive neutralizing antibodies or specific inhibitors represents a promising approach for mechanistic interrogation.

## 4. Materials and Methods

### 4.1. Bioinformatics Analysis

#### 4.1.1. Transcriptome Dataset Analysis

Dataset E-MTAB-1050 was obtained from the European Molecular Biology Laboratory’s European Bioinformatics Institute (EMBL-EBI). It contains transcriptomic profiles of neutrophils isolated from the bone marrow of C57BL/6J mice, categorized into three groups: neutrophils treated with 100 ng/mL lipopolysaccharide for 2 h (N1 group), those treated with 20 ng/mL interleukin-4 (IL-4) for 2 h (N2 group), and untreated neutrophils serving as the control group (N group) [39]. Raw sequencing data were retrieved from the European Nucleotide Archive (ENA) under accession number PRJEB45218 (https://www.ebi.ac.uk/ena/browser/view/PRJEB45218, accessed on 1 October 2025) and processed using R for data preprocessing and quality control.

#### 4.1.2. Differential Gene Expression Analysis and Functional Enrichment Analysis

Differentially expressed genes (DEGs) were identified using a threshold of |log_2_FC| > 1 and adjusted *p* < 0.05. KEGG pathway enrichment and Gene Ontology (GO) analyses—including biological processes, molecular functions, and cellular components—were subsequently performed for the comparisons N1 vs. N and N2 vs. N, incorporating the magnitude of fold changes. Statistical significance was defined as an adjusted *p* < 0.05.

#### 4.1.3. Construction of a Protein–Protein Interaction Network

The protein–protein interaction network (PPI) of DEGs was constructed using the STRING database [40], with a minimum confidence score of 0.4. The resulting network was visualized in Cytoscape 3.10.0.

#### 4.1.4. Identification of Hub Genes and Molecular Docking Analysis

The top 9 hub genes were identified using the maximal clique centrality (MCC) algorithm implemented in the CytoHubba plugin of Cytoscape 3.10.0. Homologous conversion from Mus musculus genes to their Homo sapiens orthologs was performed to facilitate cross-species analysis. The PDB file with the highest X-ray resolution was downloaded from UniProt [41] or AlphaFold [42,43] for the target protein structure. The SMILES sequence of DHA was retrieved from PubChem. Molecular docking was carried out using AutoDock software 1.5.6, following the protocol described in our previous work [44]. In brief, water molecules were removed and hydrogen atoms added to the proteins. Hydrogen atoms were also added to the small molecule, and rotatable bonds were identified. Specifically, 50 genetic algorithm (GA) runs, 3,000,000 maximum evaluations, and 30,000 generations were employed to ensure optimal conformational sampling. Molecular visualization was performed using PyMOL (v2.5.2) to identify interacting residues and hydrogen bonds [45]. The efficiency of the docking protocol was evaluated by calculating the root mean square deviation (RMSD), with a threshold of less than 2 Å, as implemented in PyMOL [46,47,48].

#### 4.1.5. Molecular Dynamics Simulation of the TNF and DHA Complex

Molecular dynamics simulations were performed using GROMACS 2022.2. The protein was described by the Amber14SB force field, and the TIP3P water model was employed for solvation. Small molecules were parameterized using antechamber to derive AM1-BCC charges and assigned atom types from the GAFF2 force field, with topologies subsequently converted to GROMACS format via ACPYPE. Ion parameters were consistent with the TIP3P model (Joung–Cheatham). The protein–ligand complex was solvated in a truncated octahedral box, ensuring a minimum distance of at least 1.2 nm between the protein surface and the box boundary. The system was neutralized by adding Na^+^/Cl^−^ ions to achieve an ionic concentration of 0.15 M. Energy minimization was carried out using the steepest descent algorithm until the maximum force fell below 1000 kJ·mol^−1^·nm^−1^. This was followed by two-stage equilibration: 200 ps of NVT and 200 ps of NPT ensemble simulations at 298 K to stabilize temperature and density. The production run was conducted under NPT conditions for 100 ns with a 2 fs time step and Verlet cutoff scheme. Long-range electrostatic interactions were treated using the Particle Mesh Ewald (PME) method, while van der Waals and short-range electrostatic interactions were truncated at 1.2 nm. Covalent bonds involving hydrogen atoms were constrained using the LINCS algorithm. Temperature and pressure were maintained at 298 K and 1 bar using the Nose–Hoover thermostat and Parrinello–Rahman barostat, respectively. Trajectories were recorded every 10 ps for subsequent analysis. Structural and interaction analyses were performed using standard GROMACS 2022.2, VMD/PyMOL 3.1.3. MM-PBSA binding free energy calculations were carried out when required using gmx_MMPBSA.

#### 4.1.6. Clustering Analysis of Hub Genes and Protein Domains

The “GOSemSim” R package was utilized to perform Friends analysis on the hub genes. Domain annotations were retrieved from the UniProt database [41] based on protein IDs, and domain enrichment analysis was conducted using the hypergeometric distribution test.

### 4.2. Cell Lines and Reagents

The human metastatic hepatocellular carcinoma cell line HCCLM3 were provided by Professor Bingdi Chen’s laboratory at Tongji University, maintained in high-glucose DMEM (Servicebio, Wuhan, China, G4515) supplemented with 10% fetal bovine serum (FBS, Gibco, Carlsbad, CA, USA, A5256501), 100 U/mL penicillin (Servicebio, Wuhan, China, G4003), and 100 μg/mL streptomycin (Servicebio, Wuhan, China, G4003). The human hepatocellular carcinoma cell line SNU387 and the human acute promyelocytic leukemia cell line NB4 were also obtained from the same laboratory and cultured in RPMI-1640 medium containing 10% FBS, 100 U/mL penicillin, and 100 μg/mL streptomycin. All cell cultures were maintained under standard conditions at 37 °C in a humidified atmosphere with 5% CO_2_.

All-trans retinoic acid (ATRA) was obtained from MCE (Monmouth Junction, NJ, UA, HY-14649). RNA extraction was finished with AG RNAex Pro RNA (Changsha, China, AG21101). Quantitative real-time PCR (qRT-PCR) reagent was obtained from Vazyme (Nanjing, China, ChamQ Universal SYBR qPCR Master Mix, Q711). Experiments were performed in triplicates. ELISA kits were obtained from Weiaobio (Shanghai, China, EH10497S, EH10269S, ET0047, EH11092S).

### 4.3. Neutrophil-like Cell Model

NB4 cells were seeded at a density of 1 × 10^5^ cells per well in 6-well plates. The control group was maintained in RPMI-1640, while the treated group was cultured in RPMI-1640 contained 1 µM ATRA. Culture medium was refreshed every two days. CD11b expression was assessed by flow cytometry (FC), and cell pellets were collected onto glass slides for Wright’s staining to morphologically validate differentiation.

### 4.4. Quantitative Real-Time PCR (qRT-PCR)

Total RNA was extracted from cells treated with DHA in different concentration for 4 h using AG RNAex Pro reagent, followed by cDNA synthesis. The primer sequences used for qRT-PCR are presented in Supplementary File. All mRNA expression levels were quantified by qRT-PCR using ChamQ Universal SYBR qPCR Master Mix (CN, Vazyme), and relative gene expression was normalized to GAPDH as an internal control.

### 4.5. Enzyme-Linked Immunosorbent Assay (ELISA)

Supernatant of dNB4 cells treated with DHA for 4 h were centrifuged at 2000× *g* for 20 min to remove debris. TNF-α, ROS, IL-1β and PD-L1 were tested in accordance with the instructions. Results were calculated based on standard curve.

### 4.6. Leukocyte-Mediated Killing Model (CKA)

The procedure referred to Cui et al. [35] HCCLM3 and SNU387 cells were seeded at a density of 5000 cells per well into 96-well plates with 200 µL of culture medium. Pretreated dNB4 cells with DHA concentrations at 0.5, 3, 5 mg/L for 4 h were co-cultured with cancer cells at a ratio of 3:1 for 24 h [35]. Then supernatant was removed, and the wells were gently washed twice with PBS. Subsequently, the CCK-8 assay was performed according to our previous work to determine the number of live cells [44].

### 4.7. Statistical Analysis

All data are expressed as mean ± standard deviation (SD). Statistical analyses were performed using GraphPad Prism 8.0. Comparisons between two groups were conducted using unpaired *t*-tests. *p* < 0.05 was considered statistically significant, marked as * *p* < 0.05, ** *p* < 0.01, *** *p* < 0.001. All experiments were independently repeated three times, with three technical replicates per experiment.

## 5. Conclusions

Our study demonstrates that tumor-associated neutrophil (TAN) subtypes possess distinct transcriptional, phenotypic, and functional profiles, with N1-like TANs exhibiting anti-tumor properties and N2-like TANs displaying pro-tumor characteristics. We identified nine key hub genes governing TAN polarization, with TNF emerging as a potential master regulator. Molecular docking analyses suggest that most of these hub proteins can stably interact with pharmacologically active compounds, supporting the feasibility of targeted modulation. Functionally, DHA promotes the polarization of neutrophil-like dNB4 cells toward the N1 phenotype, upregulating anti-tumor markers (PD-L1, NOX2), downregulating pro-tumor markers (CEACAM8), enhancing cytokine and ROS secretion, and increasing cytotoxic activity against hepatocellular carcinoma cells. These findings provide mechanistic insights into TAN plasticity and highlight DHA as a promising immunomodulatory agent for reprogramming innate immune cells, offering a potential strategy for enhancing anti-tumor immunity.

## Figures and Tables

**Table 1:** Binding affinities of DHA with hub genes.

Protein	PDB ID/Uniprot ID/AlphaFold ID	Binding Energy (kcal/mol)	Root Mean Square Deviation (Å)
TNF	5M2I	−5.96	0.11
IL1B	8RYS	−7.65	0.11
PTGS2	5F19	−6.3	0.11
CLEC10A	6PUV	−6.51	0.10
MSR1	7DPX	−6.14	0.10
BCL2A1	5WHI	−6.32	0.10
SOCS3	O14543	−7.15	0.11
ACOD1	6R6U	−5.78	0.11
CXCL16	Q9H2A7	−4.96	0.11

## Data Availability

The datasets used and/or analysed during the current study are available from the corresponding author on reasonable request. The data are not publicly available due to privacy restrictions.

## Supplementary Materials

The following supporting information can be downloaded at: https://www.mdpi.com/article/10.3390/ph19010088/s1, Table S1: Primer Sequences.
