# Supplementary material for: Dihydroartemisinin Promotes N1 Polarization of Tumor-Associated Neutrophils and Enhances Their Anti-Tumor Activity via Hub Gene Modulation
Source: Pharmaceuticals (Basel). 2026 Jan 1;19(1):88. doi: 10.3390/ph19010088 (PMC12844713; doi:10.3390/ph19010088)
Supplement: Supplementary file 1 [file pharmaceuticals-19-00088-s001.zip › pharmaceuticals-4002627-supplementary.pdf]

Supplementary Table S1. Primer Sequences

Gene	Species	Sequence
GAPDH-F	Human	GTCTCCTCTGACTTCAACAGCG
GAPDH-R	Human	ACCACCCTGTTGCTGTAGCCAA
TNF-F	Human	CTGACGAATTACAGGGCCAAT
TNF-R	Human	TGCGTGAACCTCTTGAACAGT
IL1B-F	Human	ATGATGGCTTATTACAGTGGCAA
IL1B-R	Human	GTCGGAGATTCGTAGCTGGA
PTGS2-F	Human	CTGGCGCTCAGCCATACAG
PTGS2-R	Human	CGCACTTATACTGGTCAAATCCC
CLEC10A-F	Human	GCTCCGCATACACCTGGATG
CLEC10A-R	Human	GCCGGTCGCATAGTCTGTTC
MSR1-F	Human	GCAGTGGGATCACTTTCACAA
MSR1-R	Human	AGCTGTCATTGAGCGAGCATC
BCL2A1-F	Human	TACAGGCTGGCTCAGGACTAT
BCL2A1-R	Human	CGCAACATTTTGTAGCACTCTG
SOCS3-F	Human	CCTGCGCCTCAAGACCTTC
SOCS3-R	Human	GTCACTGCGCTCCAGTAGAA
ACOD1-F	Human	GCCCTGCTTCCAACTGACTA
ACOD1-R	Human	CACCTGTGGCCTGTTGATCT
CXCL16-F	Human	CCCGCCATCGGTTCAGTTC
CXCL16-R	Human	CCCCGAGTAAGCATGTCCAC
PD-L1-F	Human	TGGCATTTGCTGAACGCATTT
PD-L1-R	Human	TGCAGCCAGGTCTAATTGTTTT
